# Redox interactions of technetium with neptunium in acid solutions

**DOI:** 10.1007/s10967-018-5908-z

**Published:** 2018-05-17

**Authors:** Maciej Chotkowski

**Affiliations:** 1 Faculty of Chemistry, University of Warsaw, Pasteura 1, 02-093 Warsaw, Poland; 2 Biological and Chemical Research Centre, University of Warsaw, Żwirki i Wigury 101, 02-089 Warsaw, Poland

**Keywords:** Technetium, Neptunium, Electrochemistry, Vis–NIR

## Abstract

Redox interaction of reduced technetium forms and technetium(VII) with neptunium(III), neptunium(IV) and neptunium(VI) have been investigated using electrochemical and spectroscopic (Vis–NIR) techniques. The neptunium species most stable in 4 M H_2_SO_4_, i.e. Np(IV) ions, do not reduce Tc(VII) in contrast to Np(VI) ions which oxidize Tc(IV) species to Tc(VII). The interaction of pertechnetates with Np(III) leads to formation of Tc(IV) species. The Vis–NIR measurements showed the generation of intermediate Tc(V) and Np(V) forms during the oxidation of Tc(IV) and competitive reduction of Np(VI). Tc(V) and Np(V) forms are characterised by the bands at 460 and 980 nm respectively.

## Introduction

Technetium and neptunium are artificial elements produced in relatively large amounts in nuclear power generation systems [1, 2]. The main technetium radioisotope presents in spent nuclear fuels (SNFs) is long living ^99^Tc (T_1/2_ = 2.13 × 10^5^ year) which is a product of beta minus decay of ^99^Mo generated as a product of fission reaction of^235^U. The ^99^Tc yield in fission products is at the level of 6% [1], which corresponds to 1 kg of Tc in 1 ton of SNF from a typical LWR (burnup of 45 GWd/tHM) [3].^237^Np (T_1/2_ = 2.14 × 10^6^ year) as the product of (n, 2n) reaction with^238^U [1, 2] is generated with a quantity of 0.40–0.48 kg in 1 ton of SNF [4]. Due to the fact that both elements are long living radioisotopes they pose a potential long term radiological risk.

Chemical properties of Tc and Np in aqueous media are complicated by the fact that both elements can exist in many oxidation states. Therefore, the redox interactions between reduced technetium species with a strongly oxidizing Np(VI) are expected to occur [5, 6] but they have not been thoroughly investigated and they are not fully explained yet. Reported schemes of technetium processes taking place in aqueous solutions in the presence of actinides (e.g. U, Np, or Pu) show a very complex redox behavior of the former which involves also unstable + V, + VI oxidation states [7–9]. Noteworthy is the fact that susceptibility of Tc(IV) species towards the oxidation strongly depends on their structure [10].

According to Rotmanov et al. [11] the dissolution of metallic Tc in an acid is accompanied by generation of reduced Tc species characterized spectroscopically by a band near 480 nm. These authors attribute this signal to Tc(V) ions. This is in line with conclusions of Maslennikov et al. [12] who reported that technetium species present in nitric acid solutions, most likely Tc(V) can be determined using a Vis band with the maximum at 480 nm. The structure of Tc(V) in concentrated sulfuric acid solutions has been investigated by Poineau et al. [13] and described as TcO^3+^ characterised by a broad band in the Vis region 650–800 nm. The other than described above technetium species were reported by Paquette [14]. These authors determined spectroscopically Tc(III) ions in slightly basic media using weak bands in Vis range at 470 (and 630) nm. This observation is in line with our results which also strongly suggest generation of Tc(III) species characterized spectroscopically by the band at 440 nm [10]. The spectroscopic properties of main Tc(IV) species present in aqueous solutions, i.e. TcO^2+^ and Tc(III/IV)-polymers [15–17].

The goal of this work was to study the redox interactions between Tc and Np species in aqueous media. In most of the papers devoted to this topic the various forms of Tc and Np were formed already in the mixture containing both elements by means of e.g. oxidation [7, 9]. Under such conditions, however, identification of investigating species is not always possible due to e.g. overlapping absorption bands of various forms of both elements. Therefore, the present work involves a separate synthesis of Tc and Np species of interest. These species were then identified and characterised by means of electrochemical and spectrophotochemical measurements.

## Experimental

Aqueous stock solutions of Tc(VII) and Np(IV) were prepared by dissolving potassium pertechnetate and neptunium dioxide in sulfuric acid solution, respectively. The concentration of TcO_4_^−^ in the prepared stock solution was 19.6 mM while the Np concentration in the stock solution was 20.3 mM. The presence of only a single form of Np species in green stock solution (Np(IV)), which was stable over a long time, was confirmed by separate spectrophotometric titrations with KMnO_4_.

### Preparation of Tc species

Technetium with oxidation states other than + 7, which are referred to as “reduced Tc” hereafter, were generated by the methods described previously [15]. The experiments were carried out by using a home made electrochemical thin layer cell with a reticulated vitreous carbon (RVC, thickness 2 mm, 100 ppi porosity, supplied by ERG Aerospace Corporation), a gold foil and an Ag/AgCl in 3 M NaCl as a working-, counter- and reference electrode, respectively. Such generated Tc forms were subsequently eluted from the pores of the working electrode (RVC) using 4 M H_2_SO_4_.

### Preparation of Np species

Two oxidation states of Np were synthesized in this study: Np(III) (as Np^3+^) and Np(VI) (as NpO_2_^2+^). Np(III) and Np(VI) species were generated electrochemically by oxidation or reduction of Np(IV) stock solution, respectively. A three electrode cell system was used with working and counter electrodes made of platinum meshes and a Ag/AgCl in 3 M KCl reference electrode. The cathodic and anodic compartments of the cell were separated by a vycor glass frit. Np^4+^ and Np^3+^ are considered as weak and strong reducing agents, respectively, while NpO_2_^2+^ is a strong oxidizer. The standard redox potential of the Np(IV)/Np(III) couple is relatively low and equals 0.155 V. Thus, one may assume that the electrogeneration of appropriate Np reduced species should not be difficult. It turned out, however, that the electrochemical procedures leading to obtaining selected Np species [18] in 4 M H_2_SO_4_ were ineffective. Therefore, low cathodic (− 2 V) and high anodic (+ 2.2 V) potentials were applied to the platinum electrode to generate of Np(III) or Np(VI) ions, respectively.

**Fig. 1:**
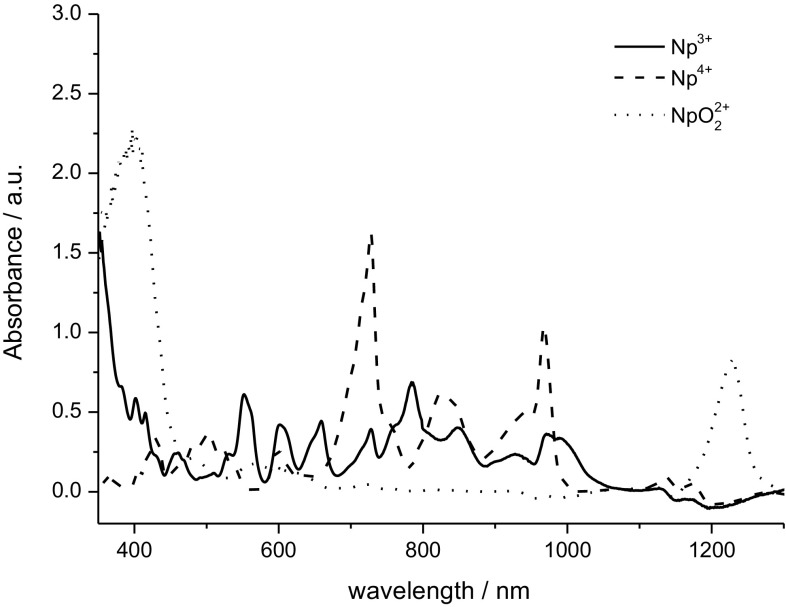
Vis–NIR spectra of 20.3 mM Np^3+^, Np^4+^ and NpO_2_^2+^ in 4 M H_2_SO_4_

Figure 1 presents the Vis–NIR spectra of Np(III), Np(IV) and Np(VI) species prepared in this study. The spectra were recorded using a Varian Cary 5 spectrophotometer. It is obvious that the shape of the spectrum clearly reflects the oxidation state of Np. That is, for Np(IV) one notes three characteristic peaks with the maxima near 730, 820 and 980 nm, NpO_2_^2+^ is characterized by a wave at 1220 nm, while for Np(III) several sharp absorption bands appear in the 500–900 nm range. The positions of all these absorption bands are in good agreements with the literature data [19]. The wavelength at 400 nm can also be attributed to NpO_2_^2+^ in the solution. The authors of Ref. [19] report a strong increase in the absorbance of Np(VI) at wavelengths below 430 nm (Fig. 2 in Ref. [19]). The lack of any signals in the range of 600–1100 nm excludes the existence of other than Np(VI) neptunium species, such as those in oxidation states of + III, + IV or + V. Further on, Np(VII) is relatively stable only in alkaline environments [6].

According to the literature data [6], + 5 is the most stable oxidation state of Np present in aqueous solutions at broad pH range. In strongly acidic media (4 M H_2_SO_4_) Np(V) ions disproportionate to Np(IV) and Np(VI) [20] according to Eq. 1:

1 $$2{\text{NpO}}_{2}^{+} + 4{\text{H}}^{+} \rightleftharpoons {\text{Np}}^{4+} + {\text{ NpO}}_{2}^{2+} + {\text{H}}_{2} {\text{O}}$$

The equlibrium constant of reaction (1) increases from 0.13 to 200 with increasing acidity from 5.34 M HClO_4_ to 8.67 M HClO_4_, respectively [21]. It is expected that the equilibrium constant of reaction (1) in sulfuric acid solutions with the same acidity as the above mentioned HClO_4_ solutions should be also relatively high due to complexation of Np^4+^ by the sulfates. The sulfates accelerate the Np(V) disproportionation [22].

### Tc–Np interaction studies

The solutions containing mixtures of as-prepared solutions of Tc and Np with different compositions were used in studies on the interaction between these radioactive elements. Cyclic voltammetry was performed to investigate the red-ox processes and stability of Tc and Np species in the mixture, their identity was evaluated on the basis of Vis–NIR measurements results.

Before starting the electrochemical measurements with the Tc-Np mixture, the mixed sample was equilibrated for 45 min. The measurements were carried out in a three electrode cell system with an Au rod, Pt mesh and Ag/AgCl in 3 M NaCl as a working, counter- and reference electrode, respectively. The anodic and cathodic compartments of the cell were separated by a vycor glass frit. Under the conditions studied, Np(VI) is expected to oxidize Tc “reduced” species while Np(III) is expected to reduce pertechnetates. In order to complete such a redox processes of Tc, the neptunium concentration was always in excess in respect to the technetium.

All the potentials mentioned hereafter are referred to the Ag/AgCl/3 M KCl reference electrode. A potentiostat/galvanostat Autolab PGSTAT101 from Metrohm was used in the electrochemical experiments.

## Results and discussion

**Fig. 2:**
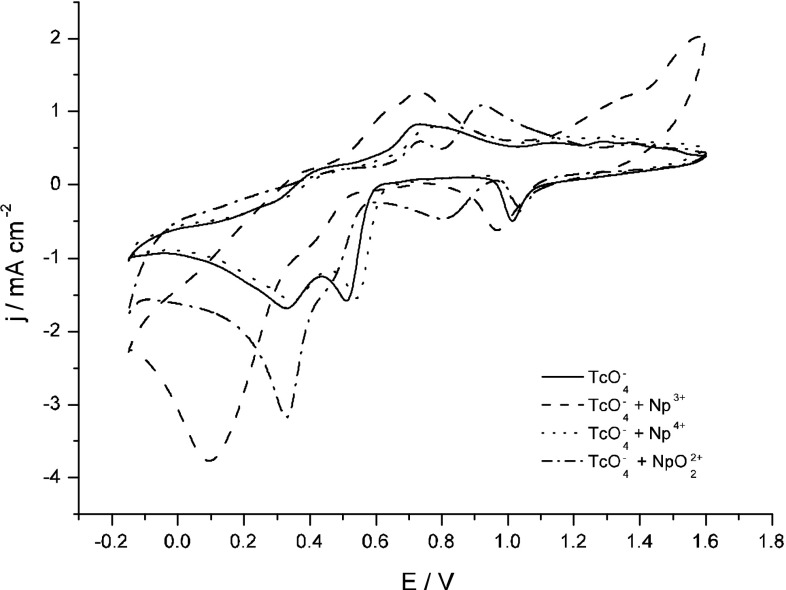
Cyclic voltammograms of 5 mM TcO_4_^−^ and 11 mM Np(III, IV or VI) in 4 M H_2_SO_4_. Au electrode, room temperature, *v* = 200 mV s^−1^

**Fig. 3:**
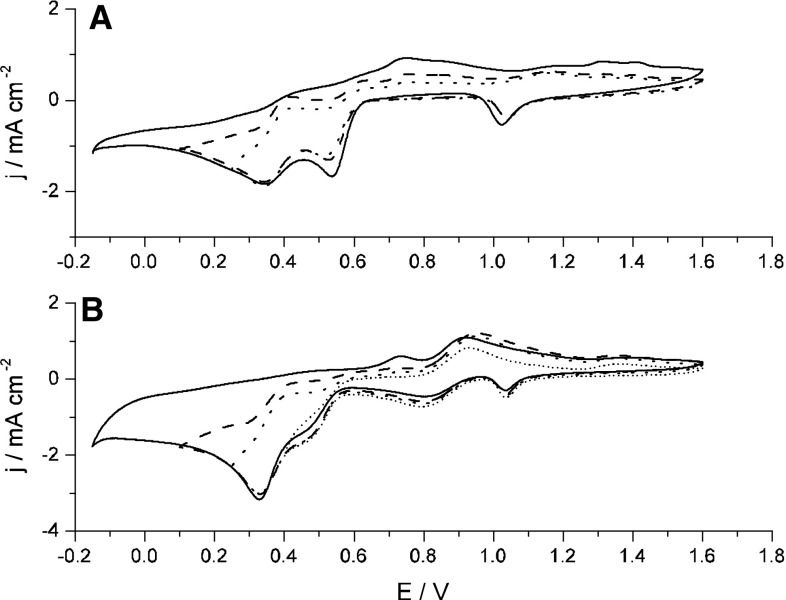
Cyclic voltammograms recorded for Au electrode in **A** 5 mM TcO_4_^−^ + 11 mM Np^4+^ + 4 M H_2_SO_4_, **B** 5 mM TcO_4_^−^ + 11 mM NpO_2_^2+^ + 4 M H_2_SO_4_ and various cathodic vertex potentials, *v* = 200 mV s^−1^

Figure 2 presents cyclic voltammograms (CVs) recorded for gold electrodes in 4 M H_2_SO_4_ containing pertechnetates with and without addition of Np(III), Np(IV) or Np(VI). In the absence of Np species (Fig. 3, solid line) the current due to pertechnetates reduction starts at ca. 0.6 V with the peak maximum at ca. 0.55 V, in agreements with previous reports [23]. This peak corresponds to the reduction of Tc(VII) to Tc(IV) via unstable Tc(V) forms [23], e.g.:

2 $${\text{Tc}}^{\text{VII}}\,{\text{O}}_{4}^{ - } + \, 6{\text{H}}^{ + } + \, 2{\text{e}}^{ - } \rightarrow {\text{Tc}}^{V} {\text{O}}^{3 + } + \, 3{\text{H}}_{2} {\text{O}}$$

3 $$3{\text{Tc}}^{\text{V}}\,{\text{O}}^{3 + } + \, 3{\text{H}}_{2} {\text{O}} \to \, 2{\text{Tc}}^{\text{IV}}\,{\text{O}}^{2 + } + {\text{ Tc}}^{\text{VII}}\,{\text{O}}_{4}^{ - } + \, 6{\text{H}}^{ + }$$

The second broad and poorly shaped cathodic wave seen at lower potentials (0–0.45 V) is due to various electrochemical and chemical reactions of technetium compounds with various oxidation states, mainly + IV and + III, i.e. electroreduction of Tc(IV) to unstable Tc(III), or possible synproportionation of Tc(III) with pertechnetates(VII). One of the possible reactions taking place in this potential range can be schematically represented as:

4 $${\text{Tc}}\left( {\text{IV}} \right) \, + {\text{ e}}^{ - }\rightarrow {\text{Tc}}\left( {\text{III}} \right)$$

5 $${\text{xTc}}\left( {\text{III}} \right) \, + {\text{ yTc}}\left( {\text{VII}} \right) \, + {\text{ zH}}^{ + } \rightleftharpoons \left[ {{\text{Tc}}\left( {{\text{III}}/{\text{IV}}} \right)_{(x + y)} {\text{O}}_{\text{q}} } \right]_{\text{poly}} + {\text{ wH}}_{2} {\text{O}}$$

Till now the mechanism of formation of polymeric Tc(III/IV or IV) is unclear. It should be noted, however, that these forms are always observed during the reduction of pertechnetates in acidic solutions and their concentration becomes greater when concentration of the pertechnatets increases.

The shape of anodic sections of voltammetric curves recorded for pertechnetates using Au electrodes strongly depends on the concentration of the former. Hence, for 0.5 mM TcO_4_^−^ investigated in our earlier studies [23] three, partially overlapped, oxidation peaks are observed while for a 5 mM pertechnetates solutions a single, poorly shaped oxidation peak is seen in potential range of 0.5–1 V (Fig. 2). A striking feature of the curves recorded for the higher concentrations of the pertechnetates is the absence of the oxidation peak at 0.4 V reported for lower TcO_4_^−^ concentration (see in [23]). This peak is attributed to the oxidation of strongly reduced Tc species to Tc(IV). Such evolution of cyclic voltammetry signals recorded for pertechnetates may be attributed to the synproportionation process takes place between Tc(III) and Tc(VII) according to Eq. 5.

The influence of Np presence on the electrochemical behavior of Tc strongly depends on the form of neptunium species. Thus, the CV curve recorded for the pertechnetates in the presence of Np(IV) (Fig. 2, dotted line) is very similar to the one recorded in the solutions containing pertechnetates only (Fig. 2, solid line). In 4 M H_2_SO_4_ the Np(IV) ions prove to be very resistant to the electrooxidation/electroreduction and do not interact with intermediate reduced technetium species generated in processes which generate currents seen on the catodic and anodic branches of CV.

Significant changes in the shape of currents due to electrochemical reactions of Tc are observed when two other forms of Np are added to the electrolyte, i.e. Np(VI) and Np(III). The strongest impact on the currents due to Tc reactions is observed for the electrolyte containing Np^3+^ (Fig. 2, dashed line). Immediately after mixing of Np^3+^ and TcO_4_^−^ solutions a red-ox reaction between Np(III) and Tc(VII) takes place. As a result, a change in the color of the solution is observed due to formation of reduced Tc. The reduction of the latter leads to formation of a huge cathodic peak at ca. 0.1 V which can be attributed to transformation of Tc(IV) into Tc in lower oxidation states.

An excess of NpO_2_^2+^ ions in respect to TcO_4_^−^ concentration has a strong impact on currents due to pertechnetates reduction (Fig. 2 dashed-dotted line). A significant decrease in the intensity of the first TcO_4_^−^ reduction peak at 0.5 V due to Np presence is noticeable.

The clear impact of Np(VI) ions on the evolution of CVs is especially visible when the cyclic voltammograms with fixed scan rate and various cathodic vertex potentials are compared (Fig. 3).

Np(VI) as a strong oxidizing agent (*E*_NpVI/NpV_^0^ = 1.136 V [6]) can oxidize intermediate technetium (V and VI) species generated in the first step of the pertechnetates electroreduction (Eq. 1), according to the Eq. 6:

6 $${\text{Tc}}^{\text{V}}\,{\text{O}}^{3 + } + 2{\text{NpO}}_{2}^{2 + } + 3{\text{H}}_{2} {\text{O}} \to {\text{Tc}}^{\text{VII}}\,{\text{O}}_{4}^{ - } + 2{\text{NpO}}_{2}^{ + } + 6{\text{H}}^{ + }$$

Reaction (6) leads to a decrease in the concentration of Tc(V) at the electrode surface. Its disappearance is manifested by a significant decrease of the intensity of the peak at 0.55 V (Fig. 3, panel B). An opposite effect is observed for the second peak of Tc reduction (at 0.35 V) which becomes higher and sharper in the presence of Np. This effect can be attributed to generation of technetium(IV). Reaction (7) is an example of this type of the process:

7 $${\text{Tc}}^{\text{VII}}\,{\text{O}}_{4}^{ - } + \, 6{\text{H}}^{ + } + \, 3{\text{e}}^{ - } \to {\text{ Tc}}^{\text{IV}}\,{\text{O}}^{2 + } + \, 3{\text{H}}_{2} {\text{O}}$$

A quasi-reversible Np(VI)/Np(V) redox system is observed at the potentials 0.6–1.1 V (Fig. 2, dashed-dotted line). This red-ox couple is characterized by the half-width potential of 0.885 V which is greater than for any known Tc(VII)/Tc(reduced species) redox system. This potential value, as well as the values of the heterogenous rate constant (0.44 cm s^−1^) and of the product of number of exchanged electrons and the transfer coefficient (0.39) are in line with the literature data for Np(V)/Np(VI) red-ox couple in sulfate solutions [24]. Obviously, all these values are not affected by the presence of pertechnetates.

**Fig. 4:**
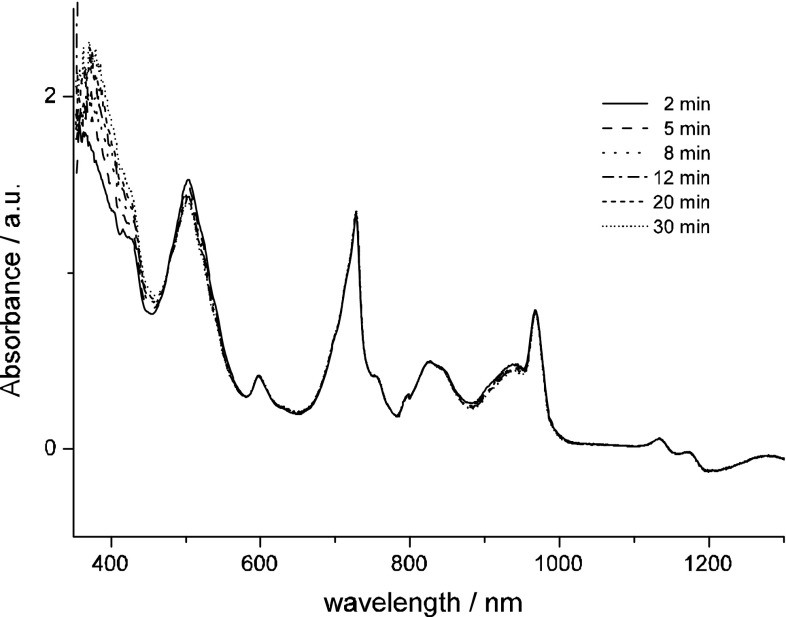
Evolution of Vis–NIR spectra recorded in 4 M H_2_SO_4_ containing 5 mM TcO_4_^−^ and 11 mM Np^3+^

The Vis–NIR experiments with Np(III)/Tc(VII) reveal that reduction of pertechnetates by Np(III) is fast and is completed within few minutes as has been shown in Fig. 4. Np(III) converts Tc(VII) ions to the polymeric Tc(IV) species characterized spectroscopically by a band at 500 nm. During this process Np(III) is oxidized to Np(IV).

The pair of redox peaks seen at 0.3–0.6 V in Fig. 3A was further investigated using a separately prepared solution which initially contained pertechnetates and Np(III) ions. The ratio of the initial concentrations of Tc(VII) and Np(III) ions (1:3) in this solution reflected the stoichiometry of the reaction (8).

8 $${\text{Tc}}^{\text{VII}}\,{\text{O}}_{4}^{ - } + \, 3{\text{Np}}^{3 + } + \, 6{\text{H}}^{ + } \rightarrow {\text{Tc}}^{\text{IV}} \,{\text{O}}^{2 + } + \, 3{\text{ Np}}^{4 + } + \, 3{\text{H}}_{2} {\text{O}}$$

**Fig. 5:**
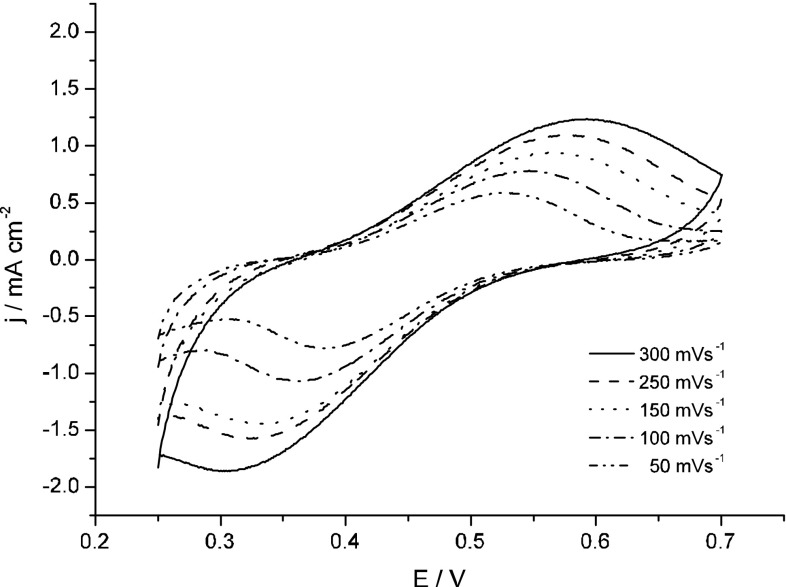
Cyclic voltammograms recorded in 3.6 mM TcO_4_^−^ + 11 mM Np^3+^ + 4 M H_2_SO_4_, with Au electrode and various scan rates

Under such conditions the Tc(VII) are completely reduced to Tc(IV) by Np^3+^ ions and the electrolyte is free of pertechnetates as long as the anodic vertex potential of the working electrode is kept too low as for electrochemical formation of Tc(V) and regeneration of Tc(VII). When the Tc(V) species appear, one may expect generation of pertechnetates in purely chemical reactions, such as disproportionation of Tc(V). The voltammetric curves recorded in such prepared electrolyte are shown in Fig. 5. The Np^4+^ species formed in reaction (8) are electrochemicaly inactive in the investigated potential range and the observed faradaic currents are attributed exclusively to the reactions of Tc species. Although the pair of redox peaks seen in Fig. 5 is apparently similar to that observed in Fig. 3a at a similar potential range the both pairs of peaks differ in respect to the peaks separation. The distance between anodic and cathodic peaks seen in Fig. 5 is much greater than in the case of Fig. 3a and strongly exceeds 0.1 V. Clearly, at least one of the electrochemical reactions connected with the redox peaks seen in Fig. 5 is not the same as in the case of the peaks observed in 0.3–0.4 V range in Fig. 3A. The latter currents are attributed to quasi-reversible or reversible reactions of Tc(III/IV)/Tc(IV) redox couple. In contrast to Fig. 3A, the separation of cathodic and anodic peaks shown in Fig. 5 is too high to be classified as a reversible or even quasi reversible system [25]. It is likely then that the anodic peak seen in Fig. 5 is due to a multistep electrochemical and chemical process of formation of TcO_4_^−^ from TcO^2+^ initially present in the electrolyte. On the other hand, the reduction process which is responsible for the formation of the cathodic peak from Fig. 5 cannot be considered as a reaction reversed to the abovementioned formation of TcO_4_^−^. It can be suggested that the reduced Tc (IV) forms present in the bulk of the solution diffuse towards the electrode and are electroreduced to Tc(III) and this process leads to formation of cathodic peak in Fig. 5. In a subsequent chemical reaction such generated Tc(III) species can undergo a polymerisation process with participation of Tc(IV). As a result, the polymeric Tc(III/IV) species are produced.

**Fig. 6:**
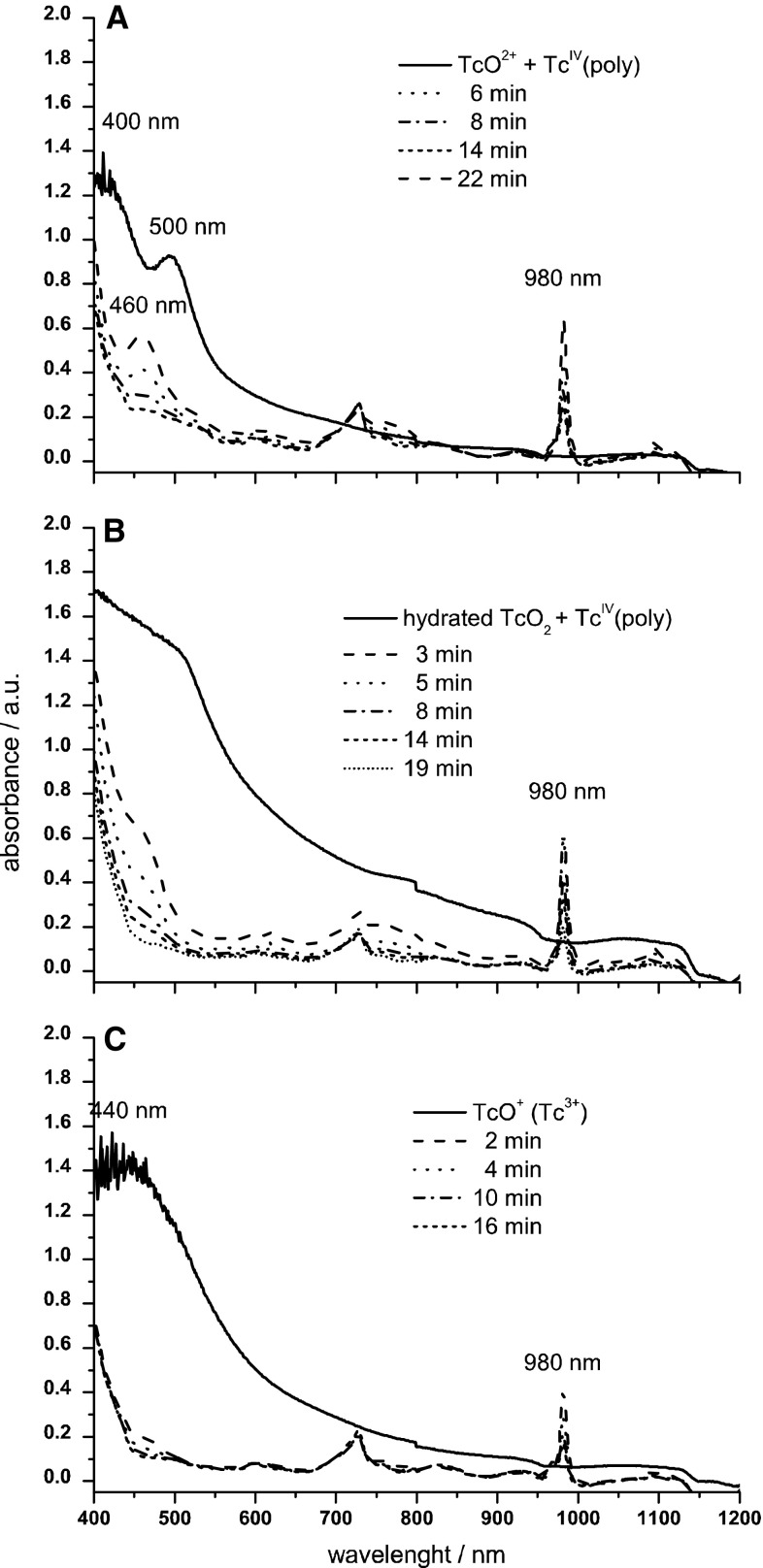
Evolution of Vis–NIR spectra recorded during redox interaction of 5 mM Tc reduced species: **A** mixture of TcO^2+^ and Tc(IV)-polymer; **B** hydrated TcO_2_ and Tc(IV)-polymer; **C** TcO^+^ (or Tc^3+^) with 11 mM NpO_2_^2+^ in 4 M H_2_SO_4_

Additional data related to the interactions between Np and Tc species were delivered from spectrophotometric experiments (Fig. 6). The experiments with Np(VI)/Tc(reduced species) reveal that oxidation of Tc(red) by Np(VI) is slower than reduction of Tc(VII) by Np(III).

The measurements carried out with the solution prepared by mixing technetium(IV) ionic species with Np(VI) (Fig. 6) show that the interaction of reduced Tc species with neptunyl(VI) ions leads to formation of intermediate Tc species characterized spectroscopically by a band with the maximum at 460 nm. Np is the only oxidizing agent present in the solution and is able to oxidize reduced Tc species with formation of the above mentioned intermediates.

The reduced Tc forms characterized spectroscopically by various bands and seen in Fig. 6 are oxidized by Np(VI). The slowest rate of this process was attained for the TcO_2_/polymeric Tc(IV) species (Fig. 6). Oxidation of all reduced Tc species leads to formation of the intermediates with the absorption band at 450–500 nm but only slow oxidation of TcO^2+^/polymeric Tc(IV) produces the intermediates observed for as long as several minutes with a well-developed band at 460 nm. Tc(V) is the possible species which can be linked with this absorption band. A very slow rate of disappearance of the Tc(V) band near 460 nm observed for TcO_2_ solution indicates that the rate of oxidation of the former species is lower as compared to Tc(III).

The Np(VI) signal at 1220 nm evolves only during initial period of ca. few minutes from the start of the reaction, later on the signal becomes apparently unchanged. For all investigated redox Tc(reduced)/Np(VI) systems the band at 980 nm appears due to formation of Np(V). This observation indicates that these ions are intermediates in the reduction of neptunyl(VI) ions. The Np(V) ions are later transformed to Np(IV) ions. The evolution of Np(VI) concentration with time is much more pronounced than for Np(V). A continuous decrease in the absorbance of Np(V) is proportional to the intensity changes of the 460 nm band of reduced Tc species. The fastest decrease of the latter band is observed for oxidation of TcO^+^ while the slowest rate is recorded during a transformation of TcO_2_/Tc(IV) polymer where the initial step (Tc(IV) → Tc(V)) requires structural changes of Tc forms.

## Conclusions

The behaviour of various technetium species in sulphuric acid solutions in the presence of Np^3+^, Np^4+^ or NpO_2_^2+^ ions has been studied. The interaction between Tc and Np species with various oxidation states was investigated in an aqueous 4 M H_2_SO_4_ by means of cyclic voltammetry and Vis–NIR spectroscopy. The results obtained in the studies allow drawing the following conclusions:

- Np(IV) is stable in a strong acidic environment, such as 4 M H_2_SO_4_, and does not interact with Tc species.
- Reduced Tc species with oxidation states lower than +VII are oxidised by Np(VI) into pertechnetates via unstable Tc(V) species. This process leads to formation of Np(IV) with Np(V) as an unstable intermediate.
- The rate of the oxidation of Tc reduced forms to pertechnetates depends on their structures and the oxidation state of Tc. Ionic forms of Tc(III) are completely oxidized to TcO_4_^−^ within few minutes in opposite to TcO_2_ or Tc(IV) polymers for which this process takes about 20 min.
